# Supplementary material for: MetaQTL: a package of new computational methods for the meta-analysis of QTL mapping experiments
Source: BMC Bioinformatics. 2007 Feb 8;8:49. doi: 10.1186/1471-2105-8-49 (PMC1808479; doi:10.1186/1471-2105-8-49)
Supplement: Additional File 7 — MetaQTL Package : jar file and tutorial. This Zip archive contains both the MetaQTL JAR file and the files of the tutorial. [file 1471-2105-8-49-S7.zip › org.inra.metaqtl/doc/org/metaqtl/main/class-use/MetaMain.html]

Uses of Class org.metaqtl.main.MetaMain


|  |  |  |
| --- | --- | --- |
| **Overview** \| **Package** \| **Class** \| **Use** \| **Tree** \| **Deprecated** \| **Index** \| **Help** | |  |
| PREV   NEXT | **FRAMES**    **NO FRAMES**     **All Classes** |  |


---


## **Uses of Class org.metaqtl.main.MetaMain**

| Packages that use MetaMain | |
| --- | --- |
| **org.metaqtl.main** |  |

Uses of MetaMain in org.metaqtl.main

| Subclasses of MetaMain in org.metaqtl.main | |
| --- | --- |
| `class` | `A2Xml` |
| `class` | `ConsMap` |
| `class` | `InfoMap` |
| `class` | `MapView` |
| `class` | `MetaDB` |
| `class` | `MMapView` |
| `class` | `MQTLView` |
| `class` | `QTLClust` |
| `class` | `QTLClustInfo` |
| `class` | `QTLModel` |
| `class` | `QTLProj` |
| `class` | `QTLTree` |
| `class` | `Xml2A` |

---


|  |  |  |
| --- | --- | --- |
| **Overview** \| **Package** \| **Class** \| **Use** \| **Tree** \| **Deprecated** \| **Index** \| **Help** | |  |
| PREV   NEXT | **FRAMES**    **NO FRAMES**     **All Classes** |  |


---
